# Unannotated single nucleotide polymorphisms in the TATA box of erythropoiesis genes show in vitro positive involvements in cognitive and mental disorders

**DOI:** 10.1186/s12881-020-01106-x

**Published:** 2020-10-22

**Authors:** Mikhail Ponomarenko, Ekaterina Sharypova, Irina Drachkova, Irina Chadaeva, Olga Arkova, Olga Podkolodnaya, Petr Ponomarenko, Nikolay Kolchanov, Ludmila Savinkova

**Affiliations:** 1 Institute of Cytology and Genetics, Siberian Branch of Russian Academy of Sciences, 10 Lavrentyev Ave, Novosibirsk, 630090 Russia; 2 Novosibirsk State University, 1 Pirogova Street, Novosibirsk, 630090 Russia; 3 Institute of Gene Biology Russian Academy of Sciences, 34/5 Vavilova Street, Moscow, 119334 Russia

**Keywords:** Erythropoiesis, Cognitive disorder, In silico prediction, TBP/TATA affinity, kinetics of TBP-TATA binding in vitro, TATA-binding protein, SNP marker

## Abstract

**Background:**

Hemoglobin is a tetramer consisting of two α-chains and two β-chains of globin. Hereditary aberrations in the synthesis of one of the globin chains are at the root of thalassemia, one of the most prevalent monogenic diseases worldwide. In humans, in addition to α- and β-globins, embryonic zeta-globin and fetal γ-globin are expressed. Immediately after birth, the expression of fetal Aγ- and Gγ-globin ceases, and then adult β-globin is mostly expressed. It has been shown that in addition to erythroid cells, hemoglobin is widely expressed in nonerythroid cells including neurons of the cortex, hippocampus, and cerebellum in rodents; embryonic and adult brain neurons in mice; and mesencephalic dopaminergic brain cells in humans, mice, and rats. Lately, there is growing evidence that different forms of anemia (changes in the number and quality of blood cells) may be involved in (or may accompany) the pathogenesis of various cognitive and mental disorders, such as Alzheimer’s and Parkinson’s diseases, depression of various severity levels, bipolar disorders, and schizophrenia. Higher hemoglobin concentrations in the blood may lead to hyperviscosity, hypovolemia, and lung diseases, which may cause brain hypoxia and anomalies of brain function, which may also result in cognitive deficits.

**Methods:**

In this study, a search for unannotated single-nucleotide polymorphisms (SNPs) of erythroid genes was initially performed using our previously created and published SNP-TATA_Z-tester, which is a Web service for computational analysis of a given SNP for in silico estimation of its influence on the affinity of TATA-binding protein (TBP) for TATA and TATA-like sequences. The obtained predictions were finally verified in vitro by an electrophoretic mobility shift assay (EMSA)*.*

**Results:**

On the basis of these experimental in vitro results and literature data, we studied TATA box SNPs influencing both human erythropoiesis and cognitive abilities. For instance, TBP–TATA affinity in the *HbZ* promoter decreases 6.6-fold as a result of a substitution in the TATA box (rs113180943), thereby possibly disrupting stage-dependent events of “switching” of hemoglobin genes and thus causing erythroblastosis. Therefore, rs113180943 may be a candidate marker of severe hemoglobinopathies with comorbid cognitive and mental disorders associated with cerebral blood flow disturbances.

**Conclusions:**

The literature data and experimental and computations results suggest that the uncovered candidate SNP markers of erythropoiesis anomalies may also be studied in cohorts of patients with cognitive and/or mental disorders with comorbid erythropoiesis diseases in comparison to conventionally healthy volunteers. Research into the regulatory mechanisms by which the identified SNP markers contribute to the development of hemoglobinopathies and of the associated cognitive deficits will allow physicians not only to take timely and adequate measures against hemoglobinopathies but also to implement strategies preventing cognitive and mental disorders.

## Background

According to World Health Organization estimates, ~ 7% of the world population have hereditary aberrations in the synthesis of hemoglobin, meaning that these are the most prevalent monogenic disorders [1]. Alpha- and β-thalassemia and sickle cell anemia are the most prevalent autosomal recessive diseases caused by mutations in the genes of α- and β-globin. β-Thalassemia was first documented in the Mediterranean, Middle-Eastern, and Asian regions, whereas sickle cell anemia was first documented in Central Africa. As a result of increasing population migration, these diseases are currently global and therefore represent a growing problem for health care in many countries. The spread of these diseases is also promoted by natural selection favoring carriers of hemoglobin synthesis aberrations who are relatively resistant to the malaria caused by *Plasmodium falciparum* [2].

It is known that human hemoglobin, the protein that carries oxygen from the lungs to various tissues, is a tetramer consisting of two α-subunits and two β-subunits of globin. Aberration one of the globin chains are the reason of thalassemia that is one of the most prevalent monogenic diseases all over worldwide [3]. Patients with severe thalassemia have serious problems related to life-long blood transfusion dependence; besides, they develop severe anemia, chronic hemolysis, and iron overload of various organs as a result of inefficient erythropoiesis, which launches compensatory mechanisms leading to, among other problems, elevated iron absorption in the heart and gastrointestinal tract [4], hepatosplenomegaly, and such complications as cardiopathy and endocrine disturbances [4]. Patients with mild thalassemia may not experience the disease manifestations and may not have anemia. α-Thalassemia, caused by deletion of one or both α-globin genes located on chromosome 16, is characterized by an almost asymptomatic clinical phenotype. The deletion of three or four genes of α-globin (Bart disease is caused by the deletion of all four genes) is characterized by a severe or lethal form of hemolytic anemia, respectively [5], where α-globin is not synthesized at all, and this condition causes fetal death (prenatal or at birth) [6]. α-Thalassemia is associated with a fairly rare syndrome of intellectual disability, ATR-X (α-thalassemia X-linked mental retardation), which features moderate or severe intellectual disability, delayed development, and other problems [7].

In addition to α- and β-globins, there are embryonic zeta-globin and fetal γ-globin in humans. Immediately after birth, the expression of fetal Aγ- and Gγ-globin stops, and then adult β-globin is expressed mostly [8].

It has been reported that in addition to erythroid cells, hemoglobin is widely expressed in nonerythroid cells including neurons of the cortex, hippocampus, and cerebellum in rodents [9]; embryonic and adult brain neurons in mice [10]; and mesencephalic dopaminergic brain cells in humans, mice, and rats [11]. Lately, there is growing evidence that different forms of anemia may be involved in or accompany the pathogenesis of various cognitive and mental disorders, such as Alzheimer’s and Parkinson’s diseases, depression of various severity levels, and bipolar disorders [12–14]. Severe depressive disorders are the third leading cause of disability worldwide [15]. Bipolar disorder is a severe psychiatric illness that starts as mild depression and brief hypomania and progresses to the acute phase with manic episodes [16].

It is known that the brain responds to a lowered hemoglobin level during anemia with vasodilation increasing cerebral blood flow. Nonetheless, when hemoglobin concentration drops substantially, the aforementioned compensatory mechanism is not triggered, and cerebral blood flow becomes insufficient, leading to brain function deficits and ischemia [17]. Both clinical [18, 19] and subclinical ischemia [20] are risk factors of mild cognitive impairment and dementia. The associations with anemias, in particular with microcytic anemia, which is most often iron deficient, may be related to oxidative stress in the brain, as a consequence of poor or ineffective iron assimilation [21]. Elevated hemoglobin levels can cause blood hyperviscosity, hypovolemia, and lung diseases, which may result in brain hypoxia and anomalies of brain function, which may also cause cognitive deficits [22]. The incidence and prevalence of anemia and hyperhemoglobinemia increase with age [23]. This state of affairs has prompted more targeted research into hematopoiesis disturbances to elucidate their mechanisms in relation to possible cognitive deficits.

Previously, by annotating experimental results from various authors, we have compiled a list of single-nucleotide polymorphism (SNP)-containing TATA boxes of human genes [24] associated with diverse hereditary monogenic diseases. The link between SNPs and diseases has been proven by those authors both molecularly and clinically. By means of the stepwise interaction of TATA-binding protein (TBP) with a TATA box [25], the sequences of TATA boxes with the surrounding nucleotides have been analyzed, and in silico predictions have been made regarding the influence of each SNP on TBP–TATA affinity. Subsequently, experimental in vitro verification of these in silico predictions has been conducted [26, 27] revealing a coefficient of correlation (r; between the prediction and experiment) of 0.822 at *p* < 10^− 7^.

After the completion of the “1000 Genomes” project [28], more than 10 thousand individual human genomes have been successfully sequenced [29], and 8.6 × 10^9^ possible SNPs have been deposited in the dbWGFP database [30]. Undoubtedly, these numerous SNPs require preliminary filtration in silico before costly and labor-intensive studies in vitro, ex vivo, and in vivo*.* With a high degree of certainty, it can be assumed that the number of identified SNPs in TATA boxes has increased substantially since 2009. These observations motivated us to update the list of previously documented TATA box SNPs in the promoters of human hemoglobin genes. Using publicly available online databases, in this work we selected unannotated SNPs of the promoters of genes associated with erythropoiesis, which were analyzed by means of our public Web service SNP_TATA_Z-tester [31], developed within the framework of the three-step biophysical model for TBP binding to TATA-like boxes [25], namely: (i) TBP slides along DNA ↔ (ii) TBP stops at a TBP-binding site [TATA-box] ↔ (iii) the TBP–promoter complex is fixed by a 90^O^ bend of DNA, − as in vitro independently observed experimentally in solution [32]. The Web service has been used by us previously to predict candidate SNP markers in TATA boxes in the context of real-world promoters of genes related to reproductive potential [33], aggressiveness [34], Alzheimer’s disease [35], obesity [36], chronopathologies [37], and autoimmune diseases [37].

In the present study, after in silico analysis of 161 unannotated SNPs, 45 candidate SNP markers were predicted for 15 out of the 25 genes participating in erythropoiesis and showing changes in TBP–TATA box affinity in the promoter. Some of them were chosen for experimental validation in vitro*.* A possible link between cognitive deficits and the influence of SNPs from TATA boxes and TATA-like sequences on the promoters of hematopoiesis genes is also discussed.

## Methods

### DNA sequences

We performed a search and retrieval of data from the databases of unannotated SNPs in the TATA boxes of erythroid genes. We employed Web service SNP_TATA_Z-tester [31], developed within the framework of the stepwise model for TBP binding to TATA-like boxes [25]. Using this software, we analyzed unannotated substitutions in the TATA boxes of human hemoglobin genes (*HBZ*, *HBB*, *HBD*, and *HBG1*) and the genes encoding the enzymes participating in heme biosynthesis (*ALAS1*, *CA1*, *EPOR*, and *GYPC*) and other proteins (involved in erythropoiesis): a total of 161 SNPs from 25 human genes. These data are publicly available thanks to Web service UCSC Genome Browser [38]. Computational analysis of 8.58 × 10^9^ possible SNPs throughout the whole human genome, predictions, experimental data, and the relevant clinical findings accumulated in database dbWGFP may accelerate and orient the clinical search for biomedical markers [30].

Genes directly participating in the processes of proliferation, differentiation, and maturation of erythrocytes in humans were retrieved from database ESGR-TRRD (erythroid-cell–specific regulated genes in TRRD format), created by Podkolodnaya O.A. and Stepanenko I.L. (http://wwwmgs.bionet.nsc.ru/mgs/papers/podkolodnaya/esg-trrd) for humans, chickens, rabbits, mice, rats, and zebrafish. The numbers of promoters, their sequences in the region [− 100; − 1] relative to the transcription start site in the 22 erythroid genes under study as well as the presence or absence of TATA boxes in these genes were determined in the Eukaryotic Promotor Database (EPD). Physical coordinates of each promoter were found in database UCSC (build 38 or GRCh38/hg38) via the BLAST software (rapidly aligning sequences to the genome). The SNPs present in promoters were retrieved from the UCSC database (build 38 or GRCh38/hg38) via a resource called Browser (to download data from the Genome Browser database). Sequence data for the substitutions and their populational characteristics were obtained from database dbSNP [39] (Database of Single Nucleotide Polymorphisms) at the National Center for Biotechnology Information (NCBI; Bethesda, MD, USA).

### DNA sequence analysis in silico

We analyzed DNA sequences between nucleotide positions − 100 and − 1 upstream of the protein-coding regions in the human genes retrieved from the human reference genome using our Web service SNP_TATA_Z-tester.

### Synthetic double-helical deoxyoligonucleotides (ODNs)

The ODNs corresponding to ancestral and minor alleles of the selected SNPs were synthesized and purified (Biosset, Novosibirsk, Russia).

### Labeling of the ODNs with ^32^P

The ODNs were 26 bp long; they were synthesized and additionally purified by polyacrylamide gel (PAAG) electrophoresis at Biosset (Novosibirsk). To prepare a labeled double-stranded ODN, both strands were labeled with ^32^P-ATP (Biosan, Novosibirsk) by means of Т4 polynucleotide kinase (SibEnzyme, Novosibirsk), annealed at 95 °C (in the equimolar ratio), and were slowly cooled (no less than 3 h) to room temperature. The annealed duplexes were purified and analyzed by electrophoresis in a 15% PAAG under nondenaturing conditions with subsequent autoradiography on a Molecular Imager PharosFX Plus phosphorimager (Bio-Rad) [40]. Unlabeled duplexes were prepared in the same way and were used after the additional purification by electrophoresis.

### Preparation and purification of recombinant full-length human TBP

In this study, recombinant human TBP (hTBP) was employed that was expressed in *Escherichia coli* BL21(DE3) cells from plasmid pAR3038-hTBP (kindly provided by Prof. B. Puhg, Center for Gene Regulation, Department of Biochemistry and Molecular Biology, The Pennsylvania State University, University Park, PA, USA). Transformation of *E. coli* BL21(DE3) was performed as described elsewhere [41]. Expression and purification of TBP were carried out as described previously [42], except for isopropyl β-d-1-thiogalactopyranoside (IPTG) concentration (1.0 instead of 0.1 mM) and induction duration (3.0 instead of 1.5 h).

### Determination of the association and dissociation rate constants of TBP–ODN complexes

Experiments on TBP–ODN binding were conducted at 25 °C in a buffer consisting of 20 mM HEPES-KOH (pH 7.6), 5 mM MgCl_2_, 70 mM KCl, 1 mM dithiothreitol, 100 μg/ml BSA, 0.01% of NP-40, and 5% of glycerol with a fixed concentration of active TBP (usually 0.5 nM).

To determine the kinetic constants of the association and dissociation of complexes, at least three independent experiments were conducted. Each experiment on kinetics usually included 32 binding reactions (eight time points, each with four concentrations of an ODN). All four binding reactions (one time point) were started simultaneously by the addition of TBP into the reaction mixture containing an ODN, with immediate transfer to a thermostat at 25 °C. Upon completion of the binding reaction, all the reaction mixtures were applied to a PAAG concurrently at electric-field intensity of 10 V/cm. TBP–ODN complexes were separated from the unbound ODN by an electrophoretic mobility shift assay. The electrophoresis was performed in a native 5% PAAG based on Tris-glycine buffer (pH 8.3) for 40 min at 10°С and electric-field intensity 25 V/cm. The gels were dried and used for exposure of a screen, Imaging Screen-K (Kodak), on the Molecular Imager PharosFX Plus phosphorimager (Bio-Rad). The screen was scanned on the phosphorimager, and the autoradiograph was analyzed quantitatively in the Quantity One software, version 4.5.0 (Bio-Rad). Velocity constants and equilibrium constants of dissociation of TBP–ODN complexes were calculated in GraphPad Prism 5 (GraphPad Software, San Diego, CA, USA).

### Statistical analysis

A comparison of our predictions with the experimental values of changes in TBP–TATA affinity after the substitutions in TATA boxes was conducted by means of two options, “Multiple Regression” and “Nonparametrics,” in the standard software STATISTICA (Statsoft™, Tulsa, USA). Changes in K_D_ (δ) with an SNP in the TATA box were calculated as -ln [K_D_,TATA_Mut_] – (−ln [K_D_,TATA]).

## Results and *DISCUSSIONS*

### In silico analysis of human erythropoiesis genes

**Fig. 1:**
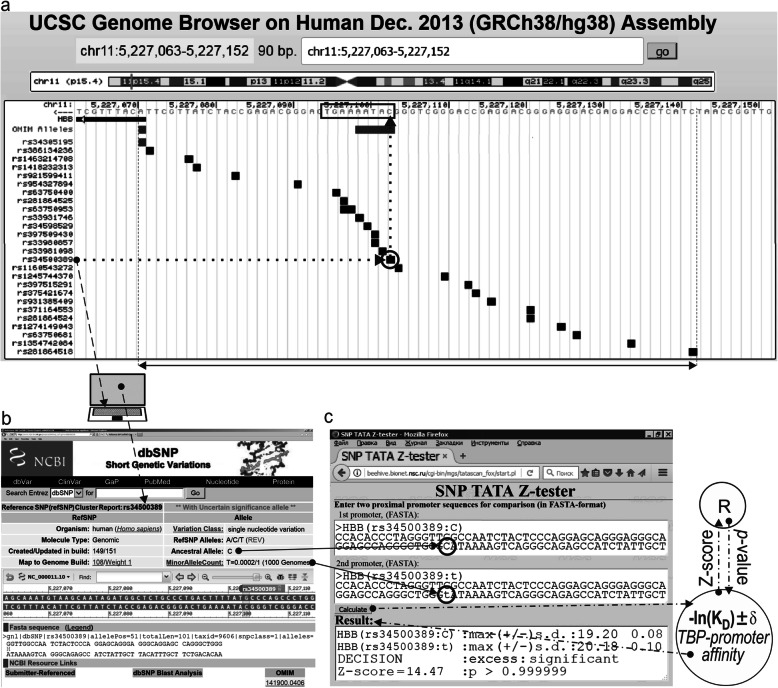
Candidate SNP marker rs34500389 predicted by this work to be associated with mental and cognitive disorders. *Legend:* **a** Unannotated SNPs (analyzed in this study) in the 100 bp region [where all known TBP-binding sites (boxed) are located; double-headed arrow, ↔] of the human *HBB* gene promoter were examined using the UCSC Genome Browser [38]. Dotted-and-dashed arrow: SNP rs34500389 under study within the reference human genome sequence and its description within database dbSNP [39], which is publicly available, as indicated by the “laptop” icon. **b** The description of rs34500389 within database dbSNP [39]. **c** The results from our Web service SNP_TATA_Z-tester [31] for SNP rs34500389 being analyzed. Dash-and-dot arrows: significance estimates for the alteration of the HBB level in patients with the minor allele (text box “2nd promoter”) relative to the norm (text box “1st promoter”) expressed as a Z-score using the R package [43]. Circles indicate the ancestral and minor alleles of the SNP marker in question within the text boxes “1st promoter” and “2nd promoter,” respectively

Tables 1 and 2 show unannotated SNPs of TATA boxes from the promoters of erythropoiesis genes; these SNPs were retrieved from publicly available databases. Predictions were made via Web service SNP_TATA_Z-tester, which is a modified version of the SNP_TATA_Z-tester Web service [31], as illustrated in the example presented in Fig. 1.

**Table 1:** Predictions of the influence of SNPs in the promoters of the hemoglobin subunit genes on TBP–TATA affinity, as performed by SNP-TATA-Z-tester [31]

Gene	Alleles, WT or mut	DNA sequence, TATA-like box; 5′-3′	Prognosis, −ln *K*_D_
*HBB*	WT rs63750400:G rs281864525:T rs34598529:G rs33980857:A rs33980857:C rs33980857:G rs33981098:C rs33981098:A rs34500389:T rs34500389:A rs33931746:C	cagggctgggcataaaactcagggcagag cagggctgggcataaaaGtcagggcagag cagggctgggcataaaTctcagggcagag cagggctgggcatGaaactcagggcagag cagggctgggcaAaaaactcagggcagag cagggctgggcaCaaaactcagggcagag cagggctgggcaGaaaactcagggcagag cagggctgggcCtaaaactcagggcagag cagggctgggcGtaaaattcagggcagag cagggctgggTataaaactcagggcagag cagggctgggActaaaactcagggcagag cagggctgggcataCaactcagggcagag	19.20 19.12 19.169 17.849 17.701 18.172 17.673 18.553 18.062 20.175 18.635 18.341
*HBB_2*	WT rs35518301:G	ggaccagcacaaaaggcagggcagag ggaccagcacGaaaggcagggcagag	19.29 18.65
*HBZ*	WT rs113180943:T rs1035033590:C	cagctccctgtaataaggggaccctg cagctccctgtaTtaaggggaccctg cagctccctgtaataaggCgaccctg	20,76 19,28 20,773
*HBZ_3*	WT rs559282746:G	ggagggtggggcccctatctctcct ggagggtggggcGcctatctctcct	17.611 17.605
*HBA1*	WT rs571582665:T	gcgccccaagcataaacctggcgcgc gcgccccaagcataaacTtggcgcgc	18.36 18.62
*HBG1*	WT rs573241527:G	agggtgcttccttttattcttcatcc agggtgcttccttttCttcttcatcc	19.06 17.54
*HBM*	WT rs905730148:A rs937192507:C	ggtgcccggaggctctataaggaggc ggtgcccggagAccctataaggaggc ggtgcccggaggcccCataaggaggc	19.13 19.23 19.95
*HBE1*	WT rs768296486:A rs1036491581:G rs150246840:C	aggcaaaaagagagcttgtgtagagc aggcaaaaagagagAttgtgtagagc aggcaaaaagagagaGtgtgtagagc aggcaaaaagagaggttgtgtagCgc	18.31 18.16 18.01 18.16

**Notes:** Ancestral (WT) and minor (mut) alleles; K_D_, dissociation constant of the TBP–DNA complex [13]; Genes: *HBB*, β-globin; *HBB*_2, β-globin promoter 2; *HBZ* zeta-globin; *HBZ*_3 zeta-globin promoter 3; *HBA1*, α1-globin; *HBG1* Aγ globin; *HBM* Hemoglobin subunit μ; and *HBE1* ε-globin

**Table 2:** Predictions regarding the influence of SNPs in the promoters of erythroid genes (except the hemoglobin subunit genes) on TBP–TATA affinity, as performed by SNP-TATA-Z-tester [31]

Gene	Alleles: WT or mut	DNA sequence, TATA-like box; 5′-3′	Prognosis, −ln *K*_D_
*ALAS1*	WT rs564394089:A rs1046254329:A rs905035347:T	ctcccgctgtatattaaggcgccggc ctacAgctgtatattaaggcgccggc ctaccgctAtatattaaggcgccggc ctaccgctatatattaaggTgccggc	20.11 20.23 20.40 20.11
*CA1*	WT rs540950375:T rs991064314:T	ggaatgggcagcttatgtacaggggg ggaatgggcaTcttatgtacaggggg ggaatgggTagcttatgtacaggggg	19.81 19.70 19.82
*CA1_2*	WT rs557418569:G rs538698304:C	cttgggcatttttatagaaacttact cttgggcGtttttatagaaacttact cttgggcattttCatagaaacttact	20.22 20.16 19.61
*HOXB2*	WT rs762972656:T	aggactccagcgaaattacagggaat aggactccaTcgaaattacagggaat	17.98 17.92
*EPOR*	WT rs1006576690:T rs971717705:C rs567946217:A rs567946217:C	cgtagcagacaaaaatagatgacgtg cgtagcagacaaaTatagatgacgtg cgtagcagacaaaaaCagatgacgtg cgtagcagacaaaaataAatgacgtg cgtagcagacaaaaataCatgacgtg	18.24 19.39 18.11 19.33 18.51
*GYPB*	WT rs3967038:C rs3967038:A	gcctactagctgttatcttccaggcc gcctacCagctgttatcttccaggcc gcctacAagctgttatcttccaggcc	18.26 18.30 18.23
*GYPC*	WT rs970970552:A rs911469201:A rs989175270:G	cattggggagttttccctgcactcct cattAgggagttttccctgcactcct cattggggagttttccctAcactcct cattggggagttttccctgGactcct	16.97 17.83 17.13 17.27
*EDRF1*	WT rs946240545:G rs774081749:G rs895690311:G	tcgcgagatttaatggcgagtcacag tcgcgagGtttaatggcgagtcacag tcgcgagattGaatggcgagtcacag tcgcgagatttaatggcgGgtcacag	18.45 18.34 17.76 18.18

**Notes:** See “Notes” under Table 1. Genes: *ALAS1* Aminolevulinate synthase 1; *CA1* Carbonic anhydrase 1; *CA1*_2 Carbonic anhydrase promoter 2; *HOXB2* Homeobox B2; *EPOR* Erythropoietin receptor; *GYPB* Glycophorin B; *GYPC* Glycophorin C; and *EDRF1* Erythroid differentiation regulatory factor 1

The cluster of human β-globin genes consists of five genes (5′-ε-γG-γA-δ-β-3′) and a locus control region [44]. Clinical manifestations of β-thalassemia (MIM 613985) mostly depend on the mutations in the regulatory and structural regions of the β-globin gene [45]. In the severe form of the disease, these manifestations consist of stunted growth, paleness, jaundice, hepatosplenomegaly, skeletal changes, and other problems as well as complications related to iron overload, including a wide range of endocrine disturbances (stunted growth, disorder of sexual maturity, diabetes mellitus and hypothyroidism, pituitary failure, and more rarely, adrenal insufficiency), dilated cardiomyopathy, and liver fibrosis and cirrhosis [46]. In patients with intermediate thalassemia, osteoporosis is present, as is the involvement of the spleen, liver, and lymph nodes as well as bone deformity, gallbladder stones, and higher susceptibility to thromboses. Mild thalassemia may be asymptomatic. It has been demonstrated that in the human genes encoding β- and δ-hemoglobins (*HBB* and *HBD*, respectively), six of many regulatory SNP markers (rs33981098, rs33980857, rs34598529, rs33931746, rs397509430, and rs35518301) weaken the affinity between TBP and the promoter of these genes and cause both thalassemia and resistance to malaria [47]. By contrast, one SNP marker (rs34500389) that enhances this affinity was found only once during the screening of newborns in Spain without any symptoms of thalassemia, i.e., this SNP allele was assumed to be harmless [48].

The zeta-globin gene (*HBZ*) codes for an α-polypeptide that is synthesized in the yolk sac of an early embryo, whereas α-globin is produced throughout the lifespan of the fetus and adult person. *HBZ* is a member of the cluster of human α-globin genes, which includes five functional genes and two pseudogenes [49]. This cluster features deletions of long segments (the most prevalent ones are the so-called Mediterranean deletion, Southeastern deletion, and Thai deletion), including α-globin genes, thereby causing α-thalassemia [50, 51]. The *Zeta2* gene is expressed at the embryonic stage and is also subject to deletion [51].

In hemoglobinopathies, mutations in DNA simultaneously influence the expression of several genes, e.g., γ-globin genes (*HBG1* and *HBG2*), responsible for the synthesis of γ-chains, which are present in fetal hemoglobin (HbF) [52]. Regulatory sequences of the 5′ region of *HBG1* and *HBG2* promoters contain at least eight SNP markers, which are located outside the core promoter region studied by us [− 100; − 1]. The main function of promoter elements of *HBG1* and *HBG2* is the binding to transcription complexes, which inhibit the expression of fetal hemoglobin at an appropriate stage [53, 54]. Unannotated rs573241527, which was detected by us, replaces Т by G in the TATA box of the *HBG1* promoter (Table 1) and is predicted to lower the affinity of the interaction with TBP.

The 5′-aminolevulinate synthase 1 gene (*ALAS1*) encodes a mitochondrial matrix enzyme, which catalyzes the first, rate-limiting stage of heme biosynthesis [55]. The most well-known role of ALAS1 is catalysis of the condensation of glycine and succinyl-coenzyme A for formation of 5′-aminolevulinate; the latter is a universal precursor of tetrapyrrole compounds participating in various reactions, including heme biosynthesis, single-electron transfer, and catalysis of redox reactions [56, 57]. It is reported that there is substantial tissue-specific expression and heme-mediated (as a feedback loop) regulation of enzymes in the heme biosynthesis pathway [8]. In particular, it is known that ALAS exists in the form of tissue-specific isozymes, that is, erythroid and nonspecific ALAS, which are encoded by two different genes. Unannotated rs1046254329 (G > A)—detected by us in the TATA box of the promoter—is predicted to slightly enhance TBP–TATA affinity, as readers can see in Table 2.

Carbonic anhydrases (CA) are zinc metalloenzymes that catalyze reversible hydration of carbon dioxide to the bicarbonate ion and proton [58, 59]. At present, there are 15 known CA isozymes belonging to the α class. CA isozymes have different distribution profiles among tissues and organs and different subcellular localizations, catalytic activities, and physiological functions [60]. The carbonic anhydrase family (CA1, − 2, and − 3) performs a multitude of functions in various species. It is known that CA1 takes part in homeostasis, mitochondrial respiration, and erythroid differentiation and may be involved in such pathological processes as anemia, chronic acidosis, and diabetic macular edema [60]. Unannotated rs538698304 detected by us in three separate projects on population research is regarded as rare: its minor allele frequencies are 0.00006, 0.0001, and 0.0002. This substitution in the middle of the TATA box of the *CA1* promoter worsens the TATA box sequence, and according to our prediction, lowers TBP–TATA affinity. Therefore, this SNP may be a candidate marker of anemia.

In addition to erythropoiesis regulation in hematopoietic tissues, erythropoietin (EPO) functions in other tissues, including the nervous system. To perform its function, EPO uses a homodimeric receptor (EPOR), which is also widely expressed in the nervous system [61]. The main role of EPOR is to stimulate the rapid spread of erythrocyte progenitors and to promote their survival [62]. Mutations of *EPOR* cause primary hereditary erythrocytosis [63] and have been detected in 15% of the cases [64]. It is well proven that the EPO/EPOR system has a wide spectrum of nonhematopoietic effects [64]. Functional receptors encoded by *EPOR* have been discovered in nonerythroid blood cell lineages, such as lymphocytes, myeloid cells, and megakaryocytes. Besides, *EPOR* is widely expressed in the central nervous system (brain) of embryos, fetuses, and adults [65]. The EPO–EPOR complex has an influence on the recovery and nutrition of brain tissues after injury [66].

The *GYPC* gene codes for two sialoglycoproteins on the erythrocyte surface in humans: glycophorin C (GPC) and glycophorin D (GPD) [67]. Parasite *P. falciparum*, which is a causative agent of malaria in humans, employs GPC to enter human erythrocytes. *GYPC* is widely expressed in the large intestine, esophagus, and 23 other organs or tissues. Studies on the molecular evolution of *GYPC* among hominids (great and smaller apes) and on polymorphism patterns at the respective locus [67] have revealed an excess of nonsynonymous between-species divergences, which apparently are related to accelerated evolution of *GYPC* in the *Homo sapiens* lineage.

DNA methylation is a hereditary epigenetic modification that stably changes the expression of genes involved in such a complex mental disorder as schizophrenia. Some researchers [68] have demonstrated that the methylation profiles of *GYPC*, which contributes to schizophrenia pathogenesis, significantly differ between female and male patients and between patients with schizophrenia and controls. Unannotated rs970970552, identified by us as a candidate SNP marker via a prediction made by means of SNP_TATA_Z-tester, increases the affinity between TBP and the TATA box of such a sequence.

### Experimental in vitro validation of the predictions about the influence of SNPs on TBP–TATA affinity

**Fig. 2:**
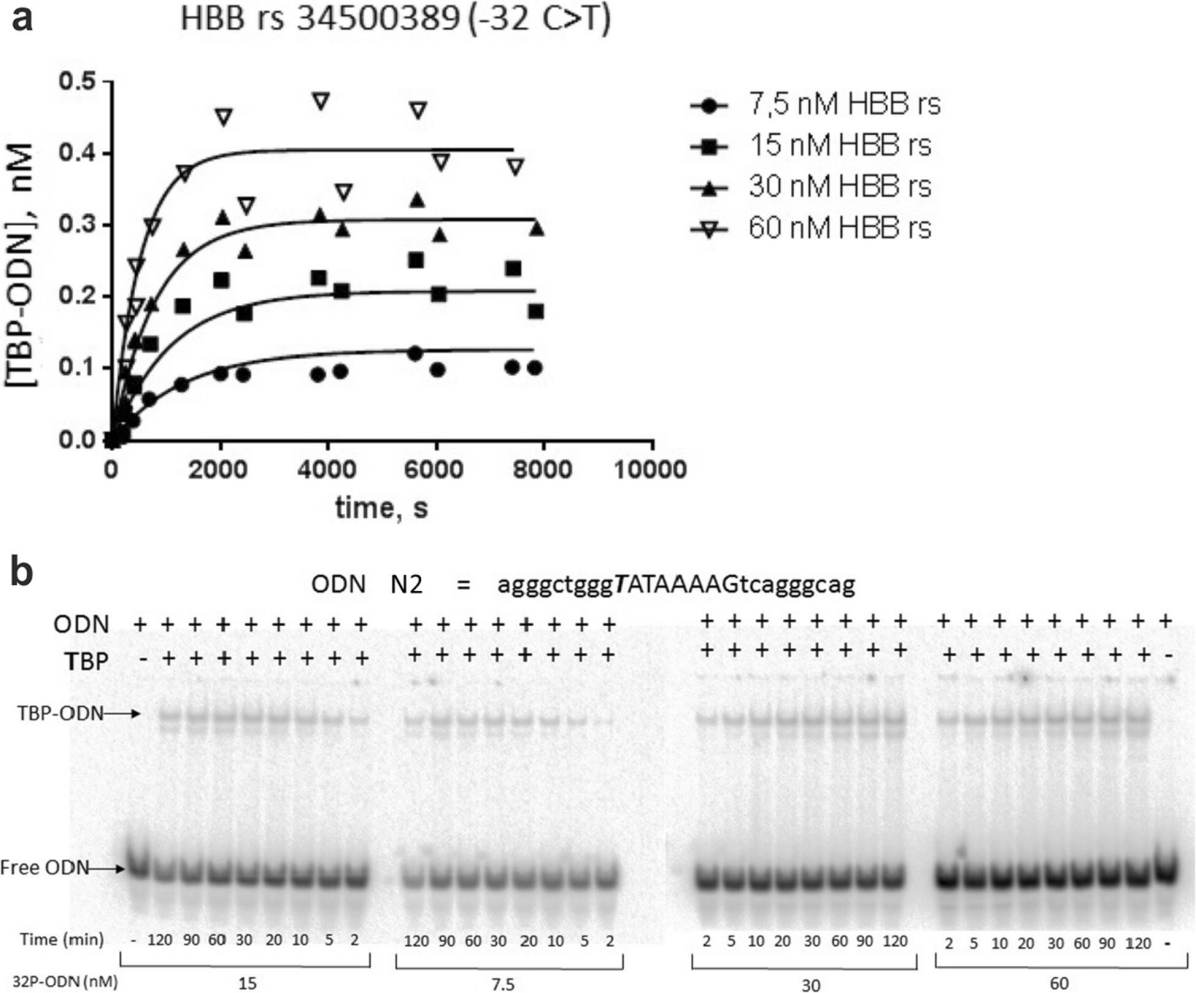
Measuring the kinetics of TBP binding to TATA-containing ODN N2 identical to the *HBB* promoter in healthy people. *Legend:* **a** Dependences of reaction rates on ODN N2 concentrations; **b** electropherograms from which these curves were derived. TBP concentration was 0.4 nM in all the experiments; the concentrations of a TATA-containing ODN that we used are indicated. TBP–TATA-associated isotherms and *k*_a_ and *k*_d_ values were inferred from the electropherograms using GraphPad Prism 5 software

For experimental in vitro validation, we chose SNP-containing TATA boxes of human genes *HBB*, *HBZ*, and *EPOR*. Table 3 presents the results of in vitro verification of our predictions regarding the influence of substitutions in TATA boxes on TBP–TATA affinity. For instance, TBP–TATA affinity in the *HbZ* promoter decreases 6.6-fold as a result of a substitution in the TATA box (rs113180943), SNPs in the TATA-like sequence of the promoter of *EPOR* (− 27 T > **A**, −31C > A, and -31C > **G**) enhancing the TBP–TATA affinity 15.5-fold, 8.1-fold, and 2.1-fold, respectively. Additionally, Table 3 shows negative natural logarithms of the predicted and experimentally determined K_D_ values and changes in K_D_ (δ) in the presence of the SNP in the TATA box: δ = *−ln [K*_*D*_*,*_*Mut*_*] – (−ln [K*_*D*_*,*_*WT*_*])*, and values of association and dissociation rate constants (*k*_a_ and *k*_*d*_, respectively) for TBP–TATA complexes, reflecting the velocities of their formation and dissociation. The results were obtained in an electrophoretic mobility shift assay (Fig. 2): dependences of reaction rates on deoxyoligonucleotide (hereinafter: ODN) concentrations – Fig. 2a; electropherograms, from which kinetic curves were derived – Fig. 2b, − as well as one can see Additional file 1: Supplementary Electropherogram.

**Table 3:** Kinetic and thermodynamic characteristics of TBP–TATA interactions

Gene	ODN	Alleles: WT or mut	Allele	Sequences (5′ – 3′ strands), 26 bp	Prediction		Experiments					
					-ln*K*_D_	δ	-ln*K*_D_	δ	*K*_D_, nM	*k*_a_, M^− 1^ s^− 1^	*k*_d_, s^− 1^	t_1/2_, min
*HbB*	1 2	WT rs34500389:T	-32c > T	agggctgggcataaaagtcagggcag agggctggg**T**ataaaagtcagggcag	19.20 20.18	+ 0.98	16.81 17.26	+ 0.45	50 ± 7 32 ± 3	(1.4 ± 0.1)*10^4^ 2.1 ± 0.1*10^4^	(7.1 ± 0.7)*10^−4^ (6.6 ± 0,5)*10^− 4^	16 ± 2 17 ± 2
*HbZ*	3 4	WT rs113180943:A	-29 t > A	agctccctgtatataaggggaccctg agctccctgta**A**ataaggggaccctg	20.76 19.28	−1.48	18.93 17.03	−1.90	6 ± 1 40 ± 20	(4.5 ± 0.4) *10^4^ (2.3 ± 0,2)*10^3^	3.0 ± 0.6)*10^−4^ (1.0 ± 0.4)*10^− 4^	38 ± 4 115 ± 15
*EPOR*	5	WT		cacgtcatctatttttgtctgctacg	18.24		15.59		170 ± 30	(2.8 ± 0,3)*10^3^	(4.6 ± 0.6)*10^−4^	25 ± 3
	6	rs1006576690:A	-27 t > A	cacgtcatctat**A**tttgtctgctacg	19.39	+ 1.15	18.33	+ 2.74	11 ± 3	(9.3 ± 0,7)*10^3^	(1.0 ± 0.3)*10^−4^	115 ± 15
	7	rs971717705:G	-29a > **G**	cacgtcatct**G**tttttgtctgctacg	18.11	−0.13	15.71	+ 0.12	150 ± 30	(2.4 ± 0,3)*10^3^	(3.6 ± 0.5)*10^−4^	32 ± 4
	8	rs567946217:A	-31c > **A**	cacgt**a**catAttttttgtctgctacg	19.33	+ 1.09	17.68	+ 2.09	21 ± 6	(6.8 ± 0.7)*10^3^	(1.4 ± 0.4)*10^−4^	82 ± 6
	9	rs567946217:G	-31c > **G**	cacgtсat**G**tatttttgtctgctacg	18.51	+ 0.27	16.34	+ 0.75	80 ± 10	(3.6 ± 0.3)*10^3^	(3.0 ± 0.5)*10^−4^	38 ± 4

ODN Oligodeoxyribonucleotide; WT Ancestral allele; mut Minor allele; *K*_D_ Equilibrium dissociation constant, which is presented as mean ± standard deviation; δ, the difference between the affinity of TBP for an ODN with and without the SNP in its TATA box expressed as natural logarithms, δ = −ln (K_D,Mut_) – [−ln (K_D,WT_)]; *k*_a_ and *k*_*d*_, association and dissociation rate constants, respectively; t_1/2 =_ ln2/k_d_, the average half-life of the TBP/ODN complexes; *HBB* β-globin gene; HBZ Zeta-globin gene; and *EPOR* Erythropoietin receptor gene

Experimental validation of the influence of the unannotated SNP (rs34500389 located in *НВВ)* changing the TATA box to a more canonical form (C-_32_ATAAAAAG → T-_32_ATAAAAAG) indicated, as predicted, an increase in TBP–TATA affinity (Table 3). This affinity for the minor allele was 1.56-fold stronger (Table 3; K_D_ = 50 nM for the ancestral allele and 32 nM for the minor allele) because of the greater (also 1.5-fold) velocity of formation of the complex. The dissociation velocity and lifetime of the complex barely changed. Perhaps the relatively small increase in affinity is explained by the presence of CATAAA in both flanks, i.e., a string of *“АТ” base pairs, which weaken the TBP–TATA binding.*

The unannotated SNP (rs113180943, detected by us in the TATA box of the *HbZ* promoter), which replaces the third Т base (having the highest weight among the nucleotides in the TATA box sequence [69]) with “А,” according to the prediction, should weaken TBP–TATA affinity. Experimental in vitro validation suggested that this substitution indeed attenuates TBP–TATA affinity 6.6-fold (*K*_D_ = 6 nM for the ancestral allele and *K*_D_ = 40 nM for -29Т > **А**). The reason is that the velocity of complex formation was 19.5-fold lower, dissociation velocity 3-fold lower, whereas the lifetime of the complex was 3-fold longer.

The promoter of the erythropoietin receptor gene (*EPOR*) contains a TATA-like sequence, ТАТТТТТG, with the weightiest “Т” at the third position [69] but with the flanks containing АТ pairs. This arrangement results in weak TBP–TATA affinity: K_D_ = 170 ± 30 nM. The -27Т > **А** substitution strengthens this affinity 15.5-fold. Of note, this enhancement of affinity is accompanied by only 3.3-fold greater velocity of formation of the complex and a 4.6-fold decrease in the velocity of its dissociation. The lifetime of the complex was more than 4-fold longer in comparison with the reference allele. In case of rs567946217 (−31С > **А**), adjacent to a TATA-like sequence upstream, affinity was 8.1-fold stronger, while the velocity of complex formation was 2.4-fold greater, the velocity of dissociation 3.3-fold lower, and complex lifetime was > 3-fold greater. The -29А > **G** substitution (rs971717705), barely affecting TBP–TATA affinity (K_D_ strengthened by 13%), caused insignificant changes in the velocities of formation and dissociation of the complex (Table 3) and most likely can be categorized as a polymorphism causing “noise.” Rs567946217 (−31С > **G**), replacing С (adjacent to the TATA box) with **G**, enhanced the affinity of the TBP–TATA binding by 2-fold, raising the velocity of formation 1.3-fold and decreasing the dissociation velocity 1.5-fold. The complex’s half-life also increased relative to the reference allele and was 38 min.

### Significant correlations between our predictions in silico and measurements in vitro

**Fig. 3:**
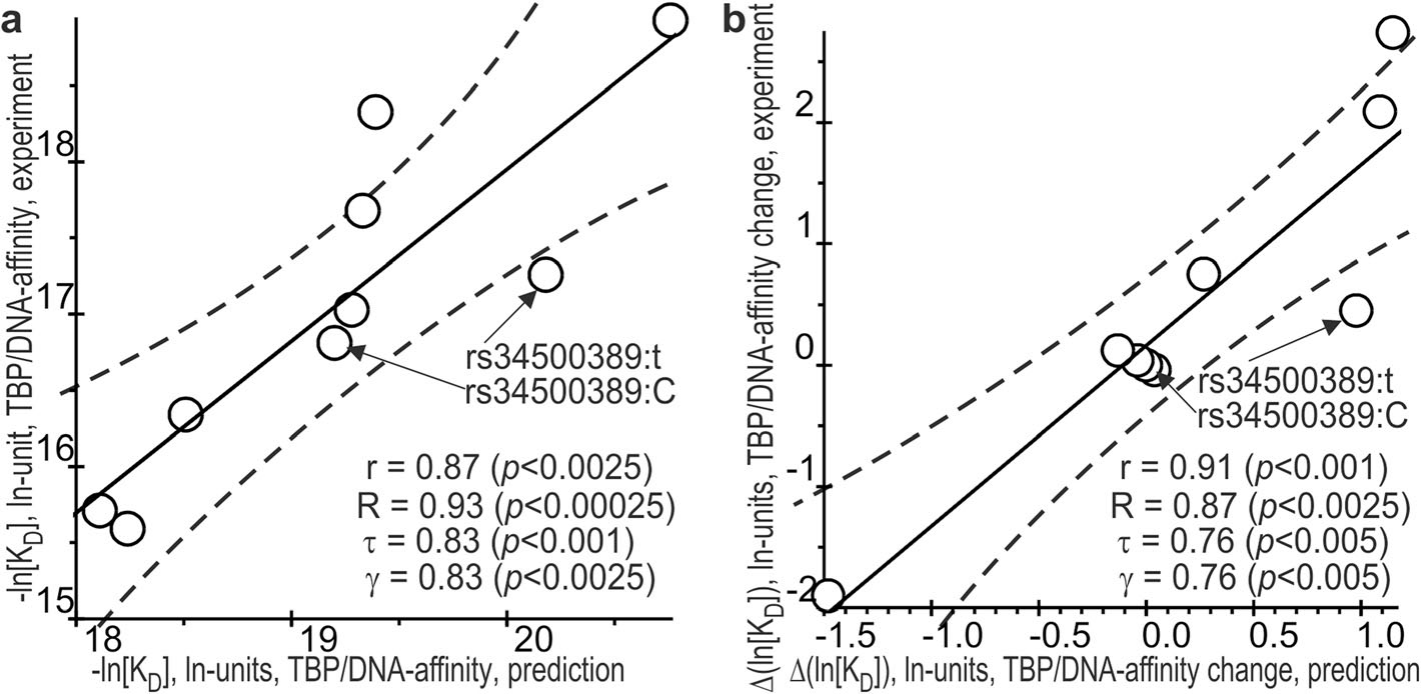
The significant correlations between the in silico predicted and in vitro experimentally measured values. *Legend:* **a** TBP–DNA affinity and **b** the TBP–DNA affinity change caused by the minor allele of the analyzed SNPs with respect to the norm, −ln [K_D_] and Δln [K_D_], respectively. Solid and dashed lines denote the linear regression and boundaries of its 95% confidence interval, calculated by means of software package STATISTICA (Statsoft™, USA); arrows pinpoint the ancestral rs34500389:C and minor rs34500389:t alleles of the SNP being studied, an analysis of which is depicted in Fig. 1 as an example of the application of our Web service SNP_TATA_Z-tester [31] in this work; r, τ, γ, and *p* are coefficients of Pearson’s linear correlation, Spearman’s rank correlation, Kendall’s rank correlation, and Goodman–Kruskal generalized correlation and their *p* values, respectively

As presented in Fig. 3, our predicted and experimental values significantly correlate with each other. As depicted in the graphs in the figure, for predicted K_D_ and its experimental values expressed in ln units (−ln [K_D_]: Fig. 3a, and predictions of measurements -ln [K_D_], δ: Fig. 3b) when the SNP was present in TATA boxes, whose values are given in Table 3, the coefficient of linear correlation r was 0.87 at *p* < 0.0025 and r = 0.91 (*p* < 0.001), respectively, whereas δ was -ln [K_D_,_Mut_] – (−ln [K_D_,_WT_]). Finally, as readers can see in Fig. 3, two considered reliable linear correlations between our predictions in silico and measurements in vitro expressed in both absolute (a) and relative (b) scales were successfully verified using three independent correlation criteria, namely: the Goodman–Kruskal generalized correlation (γ), and both Spearman’s (R) and Kendall’s (τ) rank correlations.

### A comparison between the in vitro measurements of this work with those of other authors

It should be noted that the authors of some studies have investigated the influence of a substitution of a first “Т” with “С” in a TATA box sequence. For instance, in a work dealing with the TBP of the yeast *Saccharomyces cerevisiae* [70], it was found that this substitution has almost no influence on the binding. The same authors [71] have demonstrated that a promoter containing sequence CATAAAA is 40-fold less effective at inducing transcription in vivo than the classic sequence TATAAAA. Other researchers [72] have shown that transcription in HeLa cells decreases 3-fold after a substitution of the first “Т” with “С” in the TATA box (TATAAA→CATAAA). These examples reveal that the substitution of Т with С at the first position of the TATA box has different effects on transcription and therefore on the TBP–TATA interaction. One possible reason is differences in the sequences flanking the TATA box, because in some studies [73, 74], it has been reported that the affinity of TBP for oligonucleotides differing in the number of АТ pairs in the sequences flanking the TATA box can differ 25- to 30-fold.

Our previously analyzed data on the relation of clinically confirmed diseases with substitutions in the TATA boxes of the promoter of β-globin genes indicate that the phenotype of intermediate thalassemia is associated with changes in TBP–TATA affinity causing an imbalance of chains, on average by 2.0- to 2.6-fold [26]. Consequently, carriers of the rs34500389 allele featuring 1.5-fold stronger TBP–TATA affinity may be susceptible to mild thalassemia because of the elevated amount of synthesized β-chains and can lead a normal lifestyle.

It is noteworthy that zeta-globin synthesis starts in the yolk sac in the third week of embryogenesis. By the fifth week, its level drops, and fetal ɛ-globin synthesis is initiated. Downregulation of *HbZ* after the substitution in the TATA box (rs113180943) can cause a stage-dependent disruption of “switching” events for genes of hemoglobins and the development of erythroblastosis [75]. In our study, experimental in vitro verification revealed that substitution -29Т > A (rs113180943) indeed weakens TBP–TATA affinity 6.6-fold, and accordingly, downregulates the gene. Thus, rs113180943 may be a candidate SNP marker of severe hemoglobinopathies comorbid with cognitive and mental disorders associated with disturbances of cerebral blood flow [14, 76–80]. It should be noted that the experiment coincides well with the prognosis, which is due to the TATA box sequence close to consensus. And this is especially true for “T” in the third position of TATA box, which has the maximum weight among the nucleotides of the TATA box.

As a result of studying molecular and cellular programs regulating zeta-globin synthesis, some authors [81–83] have demonstrated its good potential as a new therapeutic agent for α-thalassemia and sickle cell anemia. They found that after therapeutic reactivation, a silent but structurally intact gene of embryonic zeta-globin starts expression, and zeta-globin chains are assembled with adult β-globin into a heterotetrameric complex, Hb z2β2, fully physiologically consistent with normal adult hemoglobin [84]. This approach to α-thalassemia treatment has been illustrated on a pathological phenotype of mice with homozygous embryonically lethal deactivation of α-globin genes [82]; transgenic human zeta-globin makes these mice fully viable. Carriers of the minor allele of rs113180943 may have hemoglobinopathy in addition to the higher risk of mental disorders (more often bipolar disorder) associated with hypoxia or hyperoxia of the central nervous system [8, 14].

There is evidence that glycoprotein hormone EPO is the main regulator of erythrocyte formation and has protective properties during cerebral ischemia [85]. To perform its function, EPO binds to its receptor, EPOR, which then dimerizes and activates, thereby inducing a cascade of genes responsible for the proliferation, survival, and differentiation of erythroid progenitor cells [86]. Although in the blood, EPOR regulates erythrocyte differentiation, in the brain, it protects several types of neurons from death, including A9 dopaminergic neurons of the substantia nigra [87], and promotes oxygen storage under hypoxia; this property of EPOR is especially important for neurons with heightened needs for energy [11]. Several studies suggest that brain ischemia upregulates EPO and EPOR for repair of the damage [88, 89]. Accordingly, it can be hypothesized that SNPs in the TATA-like sequence of the promoter of *EPOR* (− 27 T > **A**, −31C > A, and -31C > **G**) enhancing the TBP–TATA affinity 15.5-fold, 8.1-fold, and 2.1-fold, respectively, in agreement with the expression levels of the gene, may indicate some degree of hereditary ischemic damage to the brain in the carriers of these alleles and may be candidate markers of this disorder. Substitutions -27 T > **A** and -31C > **A** most likely may serve as SNP markers of a rare disease, i.e., an erythropoiesis disorder [14] caused by an excessive number of erythrocytes.

The results of some experiments [77, 78] point to the participation of hemoglobins in mitochondrial respiration and metabolism. Therefore, both hypoxia and hyperoxia can damage brain tissues either by blocking ATP production or by increasing the formation of reactive oxygen species. Consequently, hemoglobins involved in mitochondrial metabolism and oxidative phosphorylation can have a major influence on neuron integrity and neurodegenerative events, which play a role in the pathogenesis of cognitive and mental disorders [14, 90]. On the other hand, SNPs -27 T > **A** and -31C > A, which cause a greater enhancement of TBP–TATA affinity and therefore upregulation of EPOR, may be candidate markers of increased risk of cognitive and mental disorders. This notion is supported by many studies indicating that both lower and higher levels of hemoglobin are related to an accelerated cognitive impairment [12, 13, 87–92].

## Conclusion

It is known that genes of hemoglobins are expressed in the astrocytes of the cortex and hippocampus and in oligodendroglia that is located in almost all brain regions, including the striatum, corpus collosum, and medulla [11]. There are many reports about the correlation between aberrations in the metabolism of hemoglobins and symptoms of mental disorders [12, 88–93]. Many investigators believe that cognitive deficits are a cause not a consequence of erythropoiesis disturbances [12, 91–97].

Thus, from previous literature data as well as our computational and experimental results, it can be concluded that the identified candidate SNP markers of erythropoiesis disturbances may also be studied in cohorts of patients with cognitive and/or mental disorders with comorbid erythropoiesis diseases in comparison to conventionally healthy volunteers. Research on the regulatory mechanisms—by which the uncovered SNP markers contribute to the development of hemoglobinopathies and the comorbid cognitive deficits—will help physicians to not only take timely and adequate measures against hemoglobinopathies but also to implement strategies preventing or slowing the progression of cognitive and mental disorders. This new knowledge is an additional resource for biomedical research, personalized medicine, diagnostics, and the development of therapeutics.

Finally, because we have already verified our in silico predictions in question using ex vivo transfections of the pGL4.10 plasmid with LUC gene-reporter (Promega, USA) into both cancer cell cultures (e.g., HCT116) [35] and fibroblast ones (e.g., hTERT-BJ1) [98], a recent cell-reprogramming technologies based on the induced pluripotent stem cell lines aimed to simulate neurodegenerations (e.g., ICGi007-A [99]) would in future give a chance for more-or-less adequate testing ex vivo the candidate SNP markers of neuropathogenesis as soon as it could be free-available and well-studied.

## Supplementary information

**Additional file 1: Supplementary Electropherogram.** The original, raw, unfiltered, uncropped, unedited electropherogram used for Fig. 2b in the cases of the minor allele T of the unannotated SNP rs34500389 of the human *HBB* gene promoter under this study.

## Data Availability

Web service SNP_TATA_Z-tester is publicly available (http://wwwmgs.bionet.nsc.ru/cgi-bin/mgs/tatascan_fox/start.pl). Due to two publicly available Web services UCSC Genome Browser (URL = http://genome.ucsc.edu/) [38] and NCBI dbSNP database build No.151 (URL = http://www.ncbi.nlm.nih.gov/snp/) [39], we predicted SNPs, which can reliably cause over- and underexpression of the 13 human erythroid genes HBB, HBZ, HBA1, HBG1, HBM, HBE1, ALAS1, CA1, HOXB2, EPOR, GYPB, GYPC, EDRF1 and IDs of which are listed, as follows: rs1006576690, rs1035033590, rs1036491581, rs1046254329, rs113180943, rs150246840, rs281864525, rs33931746, rs33980857, rs33981098, rs34500389, rs34598529, rs35518301, rs3967038, rs538698304, rs540950375, rs557418569, rs559282746, rs564394089, rs567946217, rs571582665, rs573241527, rs63750400, rs762972656, rs768296486, rs774081749, rs895690311, rs905035347, rs905730148, rs911469201, rs937192507, rs946240545, rs970970552, rs971717705, rs989175270, and rs991064314.

## Abbreviations

EMSA: Electrophoretic mobility shift assay
*k*_a_ and *k*_*d*_: Association and dissociation rate constants, respectively
K_D_ *= k*_d_ / *k*_*a*_: Equilibrium dissociation constant
ln: Natural logarithm
mut: Minor allele
ODN: Oligodeoxyribonucleotide
SNP: Single-nucleotide polymorphism
TBP: TATA-binding protein
TBP-site: TBP-binding site
t_1/2 =_ ln2/k_d_: The average half-life of the TBP/ODN complexes
WT: Wild type (norm)
δ: The difference between the affinity of TBP for an ODN with and without the SNP in its TATA box expressed as natural logarithms
*ALAS1*: Aminolevulinate synthase 1
*CA1*: Carbonic anhydrase 1
*CA1*_2: Carbonic anhydrase promoter 2
*EDRF1*: Erythroid differentiation regulatory factor 1
*EPOR*: Erythropoietin receptor
*GYPB*: Glycophorin B
*GYPC*: Glycophorin C
*HBA1*: α1-globin
*HBB*: β-globin
*HBB*_2: β-globin promoter 2
*HBG1*: Aγ globin
*HBE1*: ε-globin
*HBM*: Hemoglobin μ
*HBZ*: Zeta-globin
*HBZ*_3: Zeta-globin promoter 3
*HOXB2*: Homeobox B2
