# Supplementary figures and images for: Unannotated single nucleotide polymorphisms in the TATA box of erythropoiesis genes show in vitro positive involvements in cognitive and mental disorders
Source: BMC Med Genet. 2020 Oct 22;21(Suppl 1):165. doi: 10.1186/s12881-020-01106-x (PMC7579878; doi:10.1186/s12881-020-01106-x)

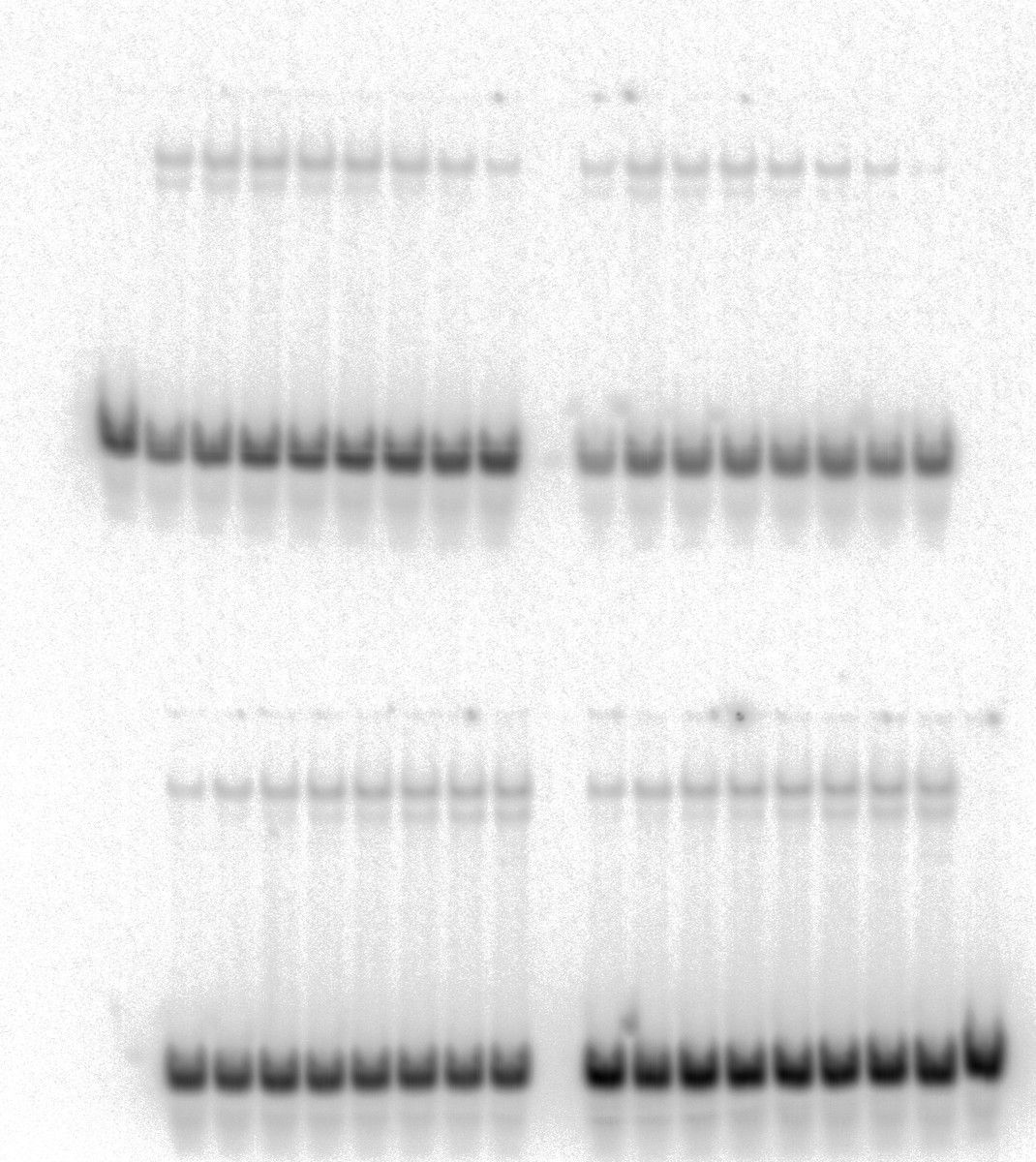

Supplement: Supplementary file 1 — Additional file 1: Supplementary Electropherogram. The original, raw, unfiltered, uncropped, unedited electropherogram used for Fig. 2b in the cases of the minor allele T of the unannotated SNP rs34500389 of the human HBB gene promoter under this study. [file 12881_2020_1106_MOESM1_ESM.tif]
